# Prescription Writing

**Published:** 1921-01-01

**Authors:** 


### Prescription Writing.

How often do we, when chance at the moment places us among the younger generation, hear the lament of our older and therefore wiser colleagues that prescription writing is a lost art? And do not many of us realise only too keenly that we have, indeed, never learned it? Some have already felt the result of this neglect; to others it has not mattered.

Nevertheless, it must be admitted that, although many a proprietary mixture is so skilfully prepared and compounded that it is hard to justify claims that it can be bettered by the average practitioner, yet, on the other hand, there is no doubt that many ready-made formulas are used simply because the physician is ignorant of how to write the necessary prescription himself. Whichever way we look at it, it is certainly a breach of the ethics of prescribing to suggest that a patient should use some preparation the precise ingredients and the main action of which are entirely unknown to the medical man concerned; yet we fear this breach is, inadvertently perhaps, committed every day of the year. This and many other essential points were recently brought forward in our contemporary the *Prescriber*, and the profession are indebted for some sound and outspoken comments.

There is, for example, the breach of merely telling the patient what to get. It requires little imagination and less actual experience of the possibilities to appreciate the danger in this procedure. Our older colleagues will say that these points are obvious and the advice unnecessary. We disagree, because any number of modern students have qualified without ever having their attention drawn to such matters. The student of to-day is the doctor of to-morrow, and there is no time to be lost in breaking the tendency to discount the practical value of good prescription writing. But let us quote the summary:

"A prescription should be carefully written on specially prepared paper. It should be written in ink, and contain the name, and probably also the address, of the patient for whom it is intended. It should always be dated. Carefully worded directions for use must be included, and the writer should initial, or far more preferably sign, it. When it contains dangerous ingredients the words 'not to be repeated' ought to be included, and last, but by no means least, it should contain neither incompatibles nor drugs the action of which is not fully known."

The regulations under D.O.R.A. by which any prescription containing certain scheduled poisons must not only be signed in full with the doctor's name and qualifications, but must also be subscribed with his address, are still broken too frequently by some members of the profession, with resulting delay and confusion.

